# Insights Into the Research Status of Neuromedin U: A Bibliometric and Visual Analysis From 1987 to 2021

**DOI:** 10.3389/fmed.2022.773000

**Published:** 2022-02-22

**Authors:** Xueping Qi, Peidong Liu, Yanjie Wang, Jinmei Xue, Yunfang An, Changqing Zhao

**Affiliations:** ^1^Shanxi Medical University, Taiyuan, China; ^2^Department of Otolaryngology, Head and Neck Surgery, Shanxi Medical University Second Affiliated Hospital, Taiyuan, China; ^3^Key Research Laboratory of Airway Neuroimmunology, Taiyuan, China

**Keywords:** Neuromedin U, H-index, bibliometric analysis, visual analysis, research status

## Abstract

Neuromedin U (NMU) is a regulatory peptide that is widely distributed throughout the body and performs a variety of physiological functions through its corresponding receptors. In recent years, NMU has become the focus of attention in various fields of research as its diverse and essential functions have gradually been elucidated. However, there have been no bibliometrics studies on the development trend and knowledge structure of NMU research. Therefore, in this study, we used VOSviewer software to statistically analyze scientific data from articles related to NMU to track the developmental footprint of this research field, including relevant countries, institutions, authors, and keywords. We retrieved a total of 338 papers related to NMU, written by 1,661 authors from 438 organizations of 41 countries that were published in 332 journals. The first study on NMU was reported by a group in Japan in 1985. Subsequently, nine articles on NMU were published from 1987 to 2006. A small leap in this field could be detected in 2009, with 30 articles published worldwide. Among the various countries in which this research has been performed, Japan and the United States have made the most outstanding contributions. Miyazato M, Kangawa K, and Mori K from the Department of Biochemistry, National Retrain and Cardiovascular Center Research Institute in Japan were the most productive authors who have the highest number of citations. Keyword analysis showed six clusters: central-nervous-system, homeostasis, energy metabolism, cancer, immune inflammation, and food intake. The three most highly cited articles were associated with inflammation. Overall, this study demonstrates the research trends and future directions of NMU, providing an objective description of the contributions in this field along with reference value for future research.

## Introduction

**Figure 1:**
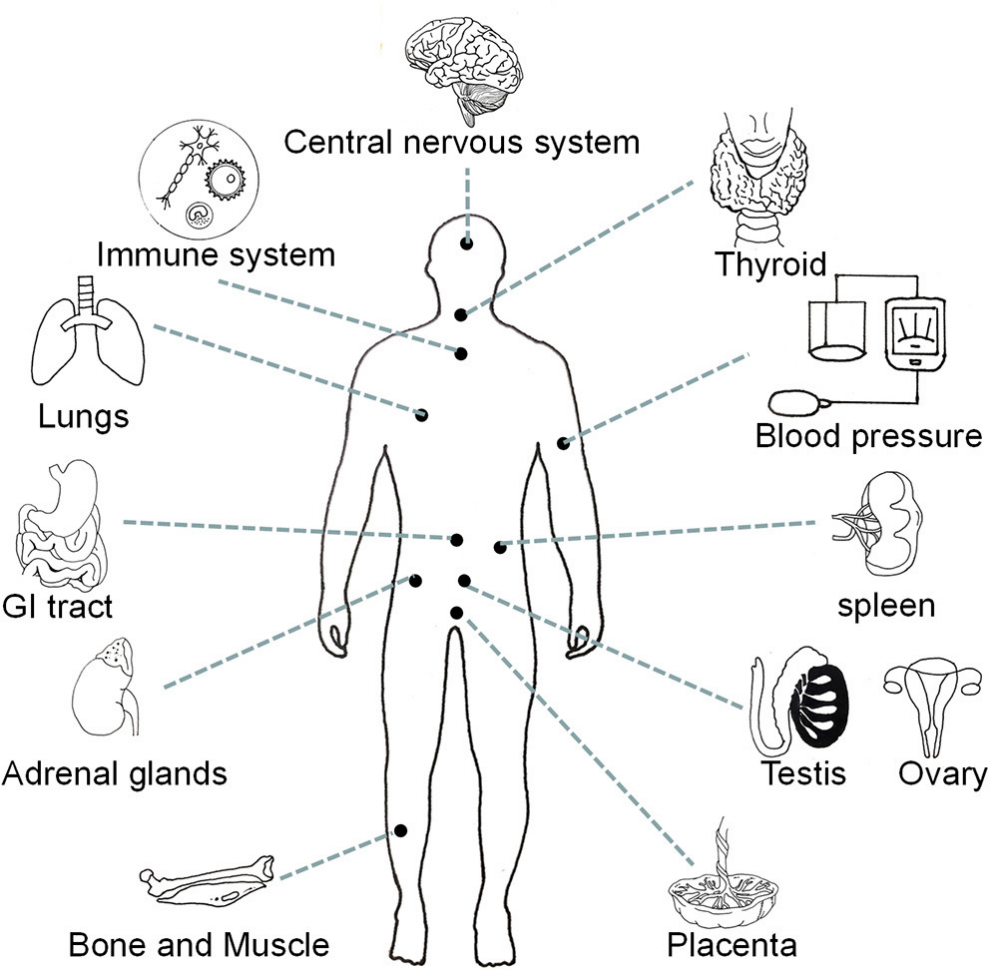
Distribution map of NMU expression in multiple organs and tissues of the human body.

Neuromedin U (NMU) is acknowledged as a highly conserved sequence neuropeptide which is a member of the neuroprotein family. The “U” in NMU derives from its ability to stimulate strong contractions in the rat uterine smooth muscle (1). Neuromedin U is secreted by different types of neurons (e.g., cholinergic, non-cholinergic, and sensory neurons), as well as by non-neuronal cells, including high expression in antigen-presenting cells (e.g., dendritic cells and monocytes) and other immune cells (e.g., B cells) (2). Neuromedin U is found in multiple parts of the body (including the testis, ovary, thyroid, spleen, placenta, and adipose tissue) and cell types (endothelial cells, keratinocytes, and lymphocytes), but is especially concentrated in the pituitary gland and gastrointestinal tract (3, 4) (Figure 1). In-depth research into the molecular mechanisms has revealed many important roles of NMU, including regulating feeding behavior, balancing energy metabolism, controlling circadian rhythms, regulating immunity, maintaining bone formation, and combating tumors and anxiety (2, 5–7).

Bibliometrics uses published data as a research object, and involves the qualitative and quantitative analysis of keywords, topics, authors, institutions, and other information in the published literature to quickly evaluate a field's research status and development trends (8, 9). Bibliometrics is used to statistically evaluate the scientific research achievements in a biomedical field, and to develop clinical practice guidelines and analyze research trends related to diseases (10). However, there have been few bibliometric reports on NMU.

Therefore, with the advantage of bibliometrics, this study provides a comprehensive and systematic analysis of the research status and development trends of NMU research, focusing on showing contributions of different regions, organizations, and authors for summary and Spatio-temporal network analysis. We present current NMU research from a new perspective and provide quantitative evaluation and trend prediction, to offer scientific guidance for researchers to clarify the value of research, seek disciplinary collaboration, and translate results.

## Materials and Methods

### Citation Data Collection

The Web of Science (WoS) database was used to find papers published in all years containing the subject term “Neuromedin U.” The final search was performed on December 16, 2021. After all data were independently extracted by the two authors, the final inclusion of the research literature was determined. The dataset was derived from original research articles, meta-analyses, reviews, and case reports. Bibliometric indices used for literature data extraction included author names, keywords, nationalities, research institutions, and citation frequencies.

### Bibliometric Analysis

We reviewed publication characteristics by generating a “WoS Literature Analysis Report” online, including countries/regions, institutions, journals, authors, and their number of annual publications, citation frequency, and “H-index.” The H-index can reflect both the quantity and quality of an author's publications, and is considered a reliable method for predicting future research success (11). Using the mapping function of the Microsoft Excel software, we compared the number of publications, citations, and H-index among different countries and institutions.

### Data Visualization Analysis

The VOSviewer software was used to visually analyze the trends in the publications (12). Author, institution, and country coupling analyses were performed using the “document coupling analysis” function, and analysis diagrams were derived. In these diagrams, the size of the circle reflects the degree of connection between published studies. Visual analysis of co-occurrence helps understand the research trends and current hot spots, which is of great significance in predicting future research dynamics (13).

## Results

### Bibliometric Analysis of NMU Research

#### Global NMU Publications Over Time

**Figure 2:**
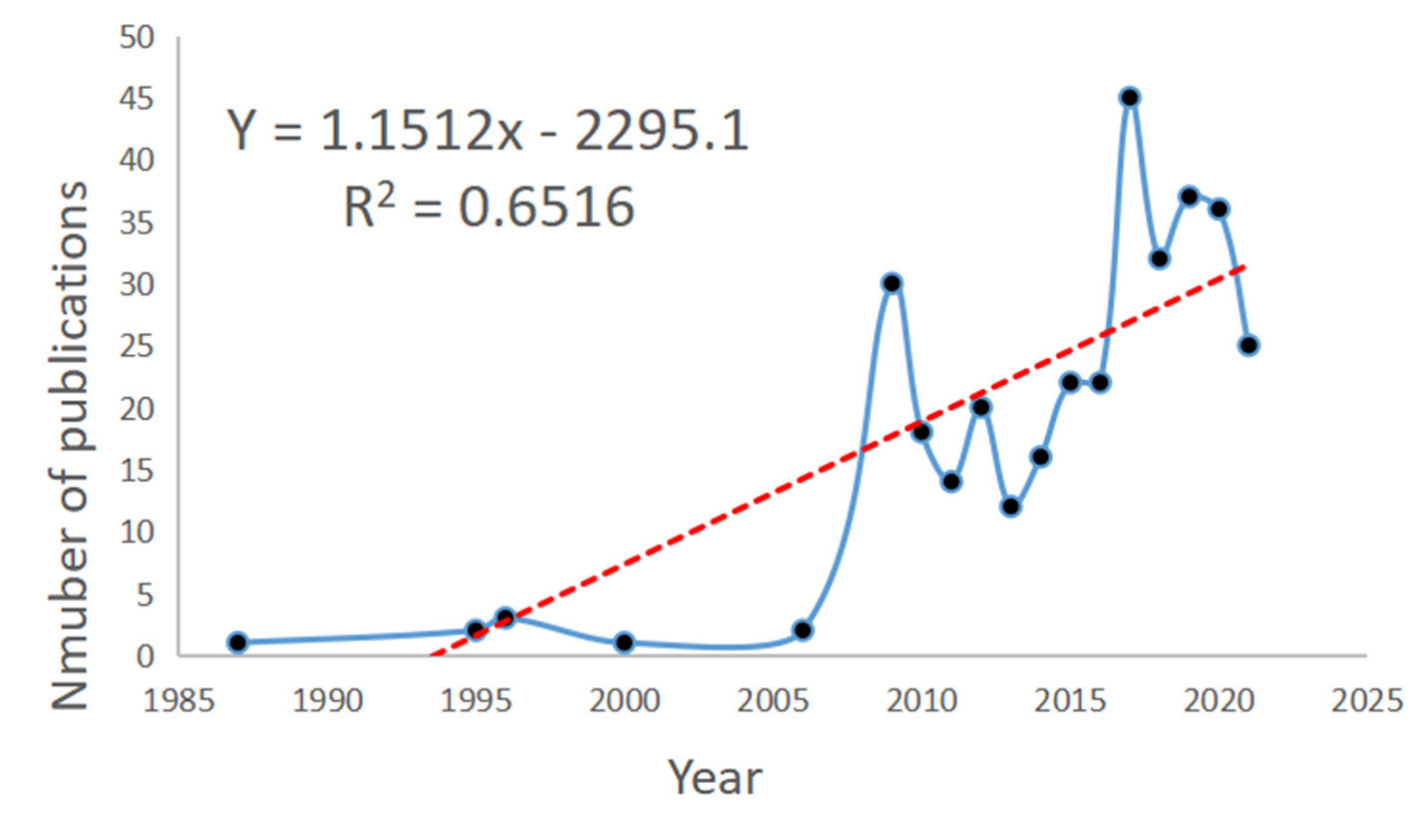
NMU publication volume over the years and the model fitting curves to predict future NMU global growth trends.

A total of 338 documents related to NMU were retrieved, including 242 research articles (71.6%), 49 reviews (14.5%), and 34 conference abstracts (10.1%; Table 1); three of the articles were published in Japanese and the rest were in English. Data analysis revealed that the first discovery of NMU was in 1985, although the first entry in the WoS database was in 1987. Between 1987 and 2006, there were few studies published on NMU. Over the next 20 years, only nine studies on NMU were published. More recently, however, the NMU research field has experienced continuous expansion. By 2009, up to 30 studies on NMU had been published. Since then, the global publication volume on NMU has experienced slight annual fluctuations, with an overall trend of steady growth, and the highest number of publications (a total of 45) was seen in 2017. Therefore, 2009 was a milestone year, in which NMU research achieved an unprecedented peak in popularity. According to the time trends, curve fitting was performed and a time prediction curve model was constructed. The equation Y = 1.1512x – 2295.1 was obtained for predicting a publication volumes and research trends for NMU in the coming years (Figure 2).

**Table 1:** The number of different types of articles in the NMU research from 1987 to 2021.

**Rank**	**Type of documents**	**Frequency**	**Percentage (%)**
1	ARTICLE	242	71.59
2	REVIEW	49	14.49
3	MEETING ABSTRACT	34	10.06
4	EDITORIAL MATERIAL	9	2.63
5	BOOK CHAPTER	4	1.18
6	CORRECTION	2	0.59
7	CONFERENCE PROCEEDINGS	2	0.59
8	PUBLISH ONLINE	1	0.29
9	NEWS ITEM	1	0.29
10	LETTER	1	0.29

#### Citation and H-Index Analysis

**Figure 3:**
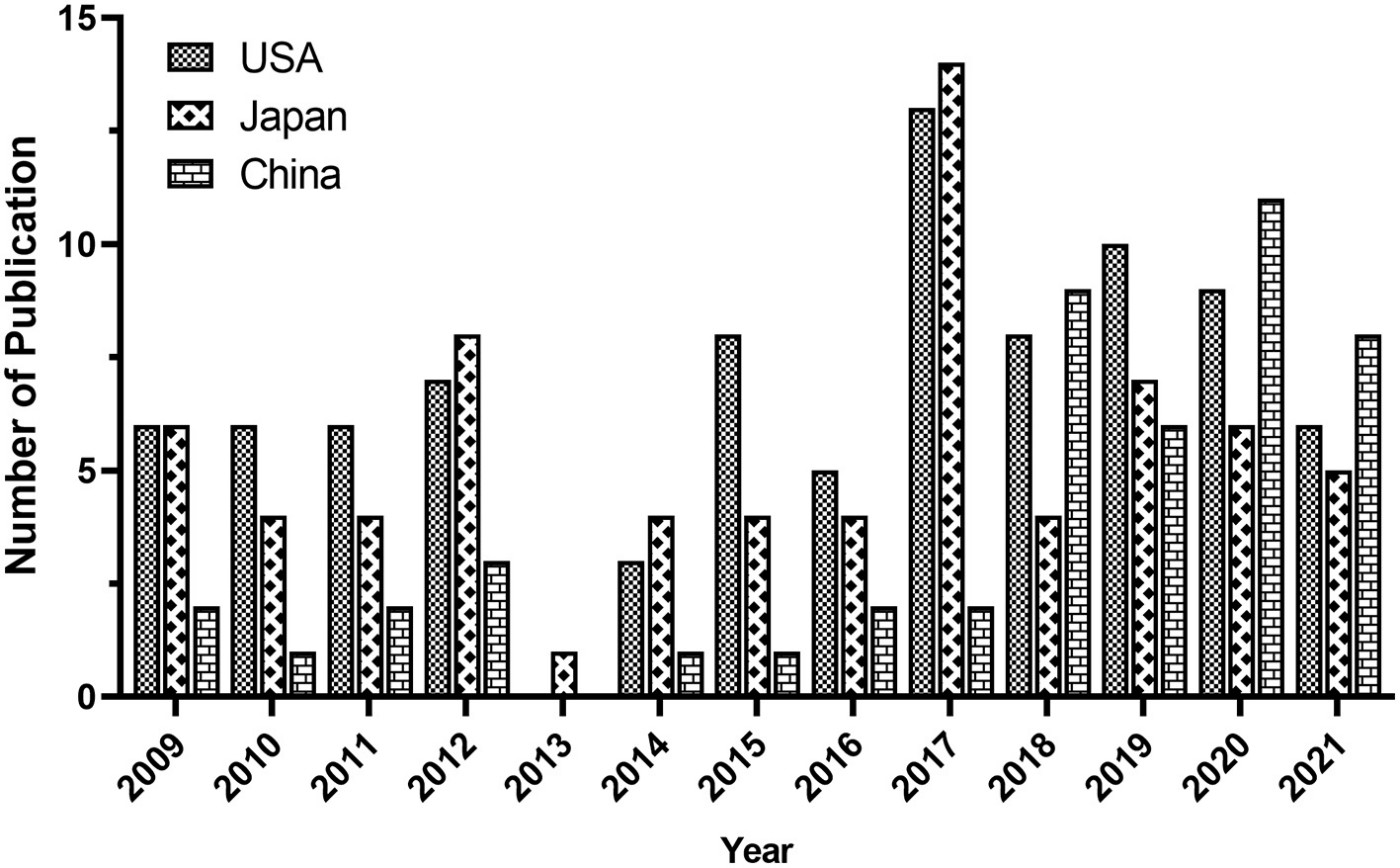
The annual amount of publications on NMU researches from the top three countries from 2009 to 2021.

According to the analysis of all 338 NMU-related documents in the WoS database, researchers in 41 countries have contributed to research progress in this field. The contributions of the top 10 countries are shown in Table 2. The United States ranked first, with the largest number of documents published (87/338, 25.74%), the highest citation frequency (2,149), and the highest H-index (14); Japan ranked second, with 80 papers published (23.67%), 777 citations, and an H-index of 15; China ranked third in the number of papers published (48/338, 14.2%), with 454 citations and an H-index of 9 (Figure 3). It is worth mentioning that all studies on NMU before 2009 were conducted in Japan.

**Table 2:** The list of top 10 countries contributed to the publications on NMU researches from 1987 to 2021.

**Rank**	**Country**	**Frequency**	**Citation counts**	**H-index**
1	USA	87	2,149	27
2	JAPAN	80	777	15
3	PEOPLES R CHINA	48	454	9
4	ENGLAND	23	665	13
5	BELGIUM	18	140	8
6	POLAND	18	119	8
7	GERMANY	17	552	9
8	ITALY	16	61	4
9	DENMARK	13	422	7
10	SWEDEN	11	108	6

#### Institutional Analysis

The number of studies published by an institution reflects its scientific research competitiveness to a certain extent. Table 3 shows the top 10 institutions in the field of NMU research globally. Four institutions were in Japan, four were in the United States, and the other two were in Sweden and Ireland, respectively. National Cerebral and Cardiovascular Center Research Institute and Miyazaki University in Japan were the top two (24, 7.10%; 20, 5.92%, respectively) and the University of Texas Medical Branch in the United States ranked thirdly (12, 3.55%). Thus, there is no doubt that institutions in these countries have made outstanding contributions to NMU research.

**Table 3:** The list of top 10 Institutions contributed to the publications on NMU researches from 1987 to 2021.

**Rank**	**Institutions**	**Country**	**Frequency**	**Percentage (%)**
1	Natl Cerebral and Cardiovasc Ctr Res Inst	Japan	24	7.10
2	Miyazaki University	Japan	20	5.92
3	University of Texas Medical Branch	USA	12	3.55
4	University of Texas System	USA	12	3.55
5	Tokyo University of Pharmacy and Life Sciences	Japan	10	2.96
6	University of California	USA	9	2.66
7	University of Gothenburg	Sweden	9	2.66
8	Cornell University	USA	8	2.37
9	Hokuriku University	Japan	8	2.37
10	University of Dublin	Ireland	8	2.37

#### Journal Publication Volume

**Figure 4:**
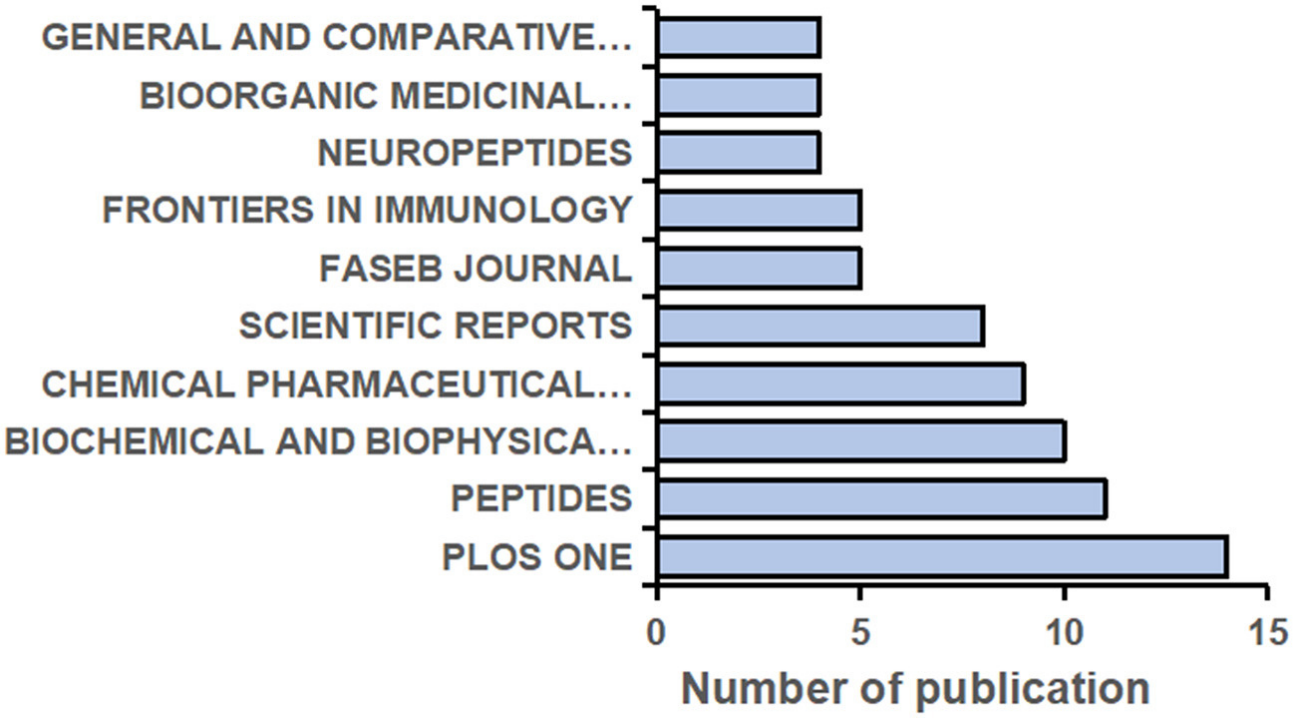
The top 10 Journals contributed to the highest volume of the publications on NMU researches from 1987 to 2021.

A total of 332 journals have published research on NMU, and the top 10 are shown in Figure 4. The top five journals each published more than six papers in this field. PLOS One [2021 impact factor (IF) = 3.24] published the largest number of studies (14 articles, 4.22%), followed by Peptides (2021 IF = 3.75; 11 articles, 3.31%) and Biochemical and Biophysical Research Communications (2021 IF = 3.575; 10 articles, 3.01%). It should be noted that the number of articles published by a journal does not represent its status in the research field but is instead related to the journal's publication cycle and the total number of papers it publishes annually. The influence of a journal should be comprehensively defined by its IF, H-index, and citation frequency together.

#### Highly Cited Papers on NMU

There were three highly cited papers on NMU which were provided online by the WoS website included in the dataset (Table 4). The three papers, all published in Nature in 2017, describe how NMU regulates group II innate lymphocytes (ILC2s) involved in inflammation.

**Table 4:** The list of highly-cited papers on NMU in the Web of Science database from 1987 to 2021.

**Rank**	**Author**	**Highly cited documents**	**Source**	**Year**	**Total cited**
1	• Wallrapp A • Riesenfeld SJ • Burkett PR	The neuropeptide NMU amplifies ILC2-driven allergic lung inflammation	Nature	2017	277
2	• Cardoso V • Chesne J • Ribeiro H	Neuronal regulation of type 2 innate lymphoid cells via Neuromedin U	Nature	2017	251
3	• Klose, CSN • Mahlakoiv T • Moeller JB	The neuropeptide Neuromedin U stimulates innate lymphoid cells and type 2 inflammation	Nature	2017	245

### Coupling Visual Analysis of NMU Research Worldwide

#### Author Coupling Analysis

**Figure 5:**
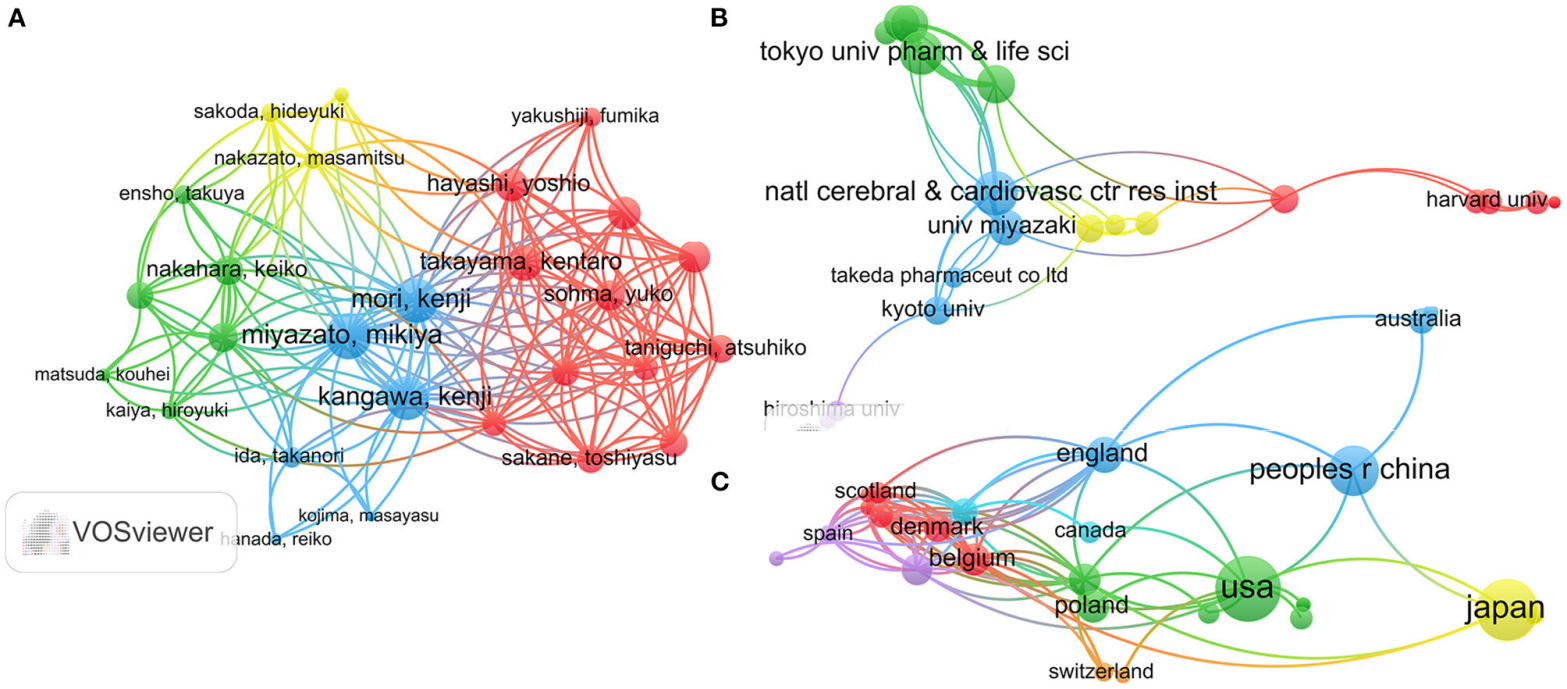
Document coupling analysis. The volume of the circle reflects the number of literatures, and the color represents the clustering. The larger the circle represents the higher frequency of occurrence. Smaller distance between two circles means more co-occurrence. The greater the density of lines between circles, the stronger the correlation. **(A)** Author coupling analysis; **(B)** organization coupling analysis; **(C)** country coupling analysis.

The top 10 authors in the field are shown in Table 5. The 338 documents retrieved had a total of 1,661 authors, 109 of whom were linked to at least three publications (Figure 5A). The top three authors, with the highest document coupling strength and the highest numbers of published articles, were Miyazato Mikiya (23 articles, coupling strength: 154), Mori Kenji (19 articles, coupling strength: 147), and Kangawa Kenji (19 articles, coupling strength: 139).

**Table 5:** The top 10 authors contributed to the publications on NMU researches from 1987 to 2021.

**Rank**	**Author**	**Frequency**	**Percentage (%)**	**H-index**
1	Miyazato M	23	6.81	8
2	Kangawa K	19	5.62	7
3	Mori K	19	5.62	7
4	Hommel JD	11	3.25	6
5	Murakami N	11	3.25	6
6	Takayama K	11	3.25	6
7	Nakahara K	10	2.96	5
8	Maruyama K	10	2.96	5
9	Hayashi Y	9	2.96	5
10	O'driscoll L	9	2.96	5

#### Organization Coupling Analysis

A total of 438 institutions were included in the dataset, and 77 were involved in at least three published studies (Figure 5B). The top three institutions for NMU research in terms of coupling strength were Ghent University (coupling strength: 26), Consiglio Nazionale delle Ricerche CNR (coupling strength: 24), and the National Institute for Health Development Estonia (coupling strength: 24).

#### Country Coupling Analysis

Scientists in 41 countries have contributed to NMU research to date, and 27 countries have published at least three papers on the topic (Figure 5C). The top five countries in terms of coupling strength were Germany (coupling strength: 46), Belgium (coupling strength: 39), Italy (coupling strength: 38), the United States (coupling strength: 36), and Hungary (coupling strength: 31). In contrast to its high ranks for publications, citations, and H-index, the United States ranked fourth in terms of connection strength. The remainder of the top five countries is in Europe, indicating close cooperation among European countries.

### Visual Co-occurrence Analysis of Global NMU Research

**Figure 6:**
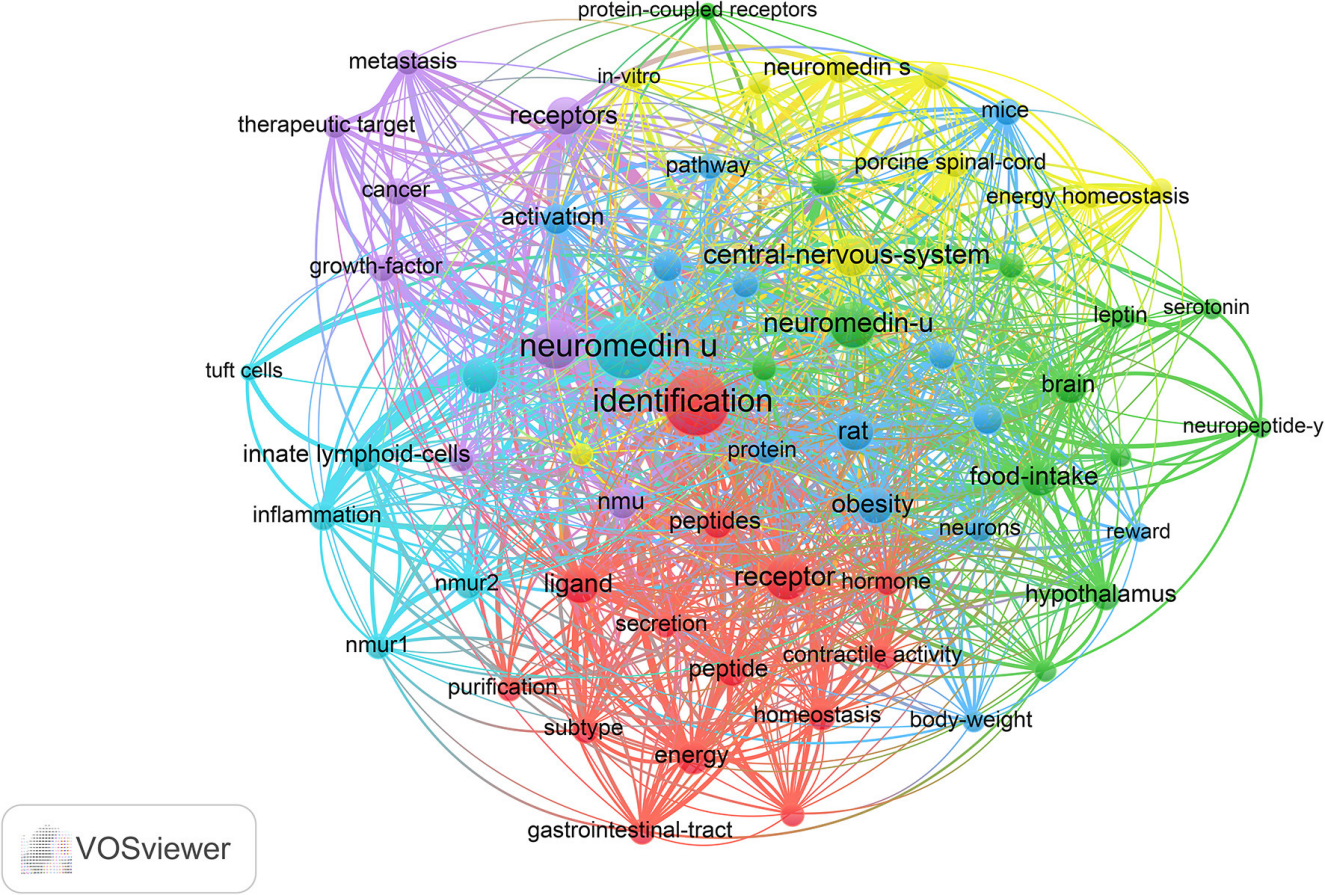
The analysis of keywords in NMU research. The keywords were classified into six clusters and were depicted in different colors (“central-nervous system,” in yellow; “homeostasis,” in red; “energy metabolism,” in blue; “cancer,” in purple; “immune-inflammatory,” in fluorescent blue, and “food intake,” in green).

According to keyword clustering, current NMU research directions could be divided into six categories: central-nervous-system, homeostasis, energy metabolism, cancer, immune inflammation, and food intake depicted by different colors (Figure 6). In the central-nervous-system, commonly used keywords included “corticotropin-releasing hormone,” “porcine spinal-cord,” “protein-coupled receptor,” “smooth-muscle,” and “central nervous system;” in the homeostasis cluster, commonly used keywords included “energy,” “homeostasis,” “secretory,” “gastrointestinal-tract,” and “hormone”; in the energy metabolism cluster, commonly used keywords included “body-weight,” “obesity,” “angiotensin,” “reward,” and “paraventricular nucleus”; for cancer cluster, commonly used keywords included “cancer,” “gene,” “metabolism,” and “therapeutic target”; in the immune-inflammatory system, commonly used keywords included “innate lymphocytes,” “tuft cells,” “inflammation”; and for food intake cluster, commonly used keywords included “leptin,” “feeding-behavior,” “glucagon-like peptide-1,” and “brain.” The keywords related to each system were both different and interrelated.

## Discussion

### Overview of NMU Research

Bibliometric analysis revealed that most NMU research to date has been basic research, and these published articles mainly focus on animal experiments and review articles, as well as a few reports on clinical trials. Although NMU research has made great progress in various aspects, clinical research on NMU remains minimal.

Neuromedin U was first discovered and isolated from the pig spinal cord by Minamino et al. (15). In 1987, the Fujii group attempted to synthesize NMU-8 and NMU-25 using the deprotecting reagent trimethylsilyl trifluoromethanesulfonate (16). Subsequently, NMU has been detected in several species, including various fish (17–19), guinea pigs (20), dogs (21), chickens (22), rabbits (23), various frogs frogs (24, 25), and humans (26), etc. Neuromedin U has maintained a highly conserved amino acid sequence during evolution, especially in its five C-terminal amino acids (-Phe-Arg-Pro-Arg-Asn), which are preserved in almost all mammals and ensure maintenance of the protein's important physiological functions (27). However, the expression levels and functions of NMU differ between tissues and species.

### NMU Versatility

Visualization of keyword co-occurrence revealed that NMU research to date has focused on six topics: central-nervous-system, homeostasis, energy metabolism, cancer, immune inflammation, and food intake. These themes are both independent and concomitant, and form an intricate network structure that reflects the versatility of NMU. Neuromedin U was first discovered based on its ability to because it can stimulate uterine contractions, and was subsequently confirmed to act as a contractile stimulator in the gastrointestinal tract and genitourinary system (14, 28). Neuromedin U has a variety of physiological functions, which are mainly related to the corresponding NMU receptors (NMURs). Typical NMURs are NMUR1 and NMUR2, both of which are of the orphan class A rhodopsin G protein-coupled receptor family (1). NMUR1 is predominantly distributed in peripheral tissues such as the gastrointestinal tract, genitourinary system, pancreas, bone marrow, lymphatic tissue, and lungs; therefore, it mainly mediates the physiological functions of NMU in peripheral tissues (1, 27). Conversely, NMUR2 is expressed in the central nervous system (mainly in the medulla oblongata, pontine network, anterior pituitary, spinal cord, and thalamus), and therefore mediates the role of NMU at these sites (27, 29). In other tissues, NMUR1 and NMUR2 are co-expressed.

As a multifunctional neuropeptide, NmU plays a variety of roles in different cells and tissues, transmitting central signals and directly stimulating tissues or cells to perform its jack-of-all-trades function. For the six themes mentioned above, a great deal of research has been carried out by scholars in different fields. Neuromedin U selectively regulates behaviors in different areas of the brain. For example, when NMU is injected into the nucleus accumbens shell, it can prevent acute alcohol-induced motor stimulation behavior and induce memory recovery as part of the alcohol reward pathway (30). Furthermore, a recent study found that NMUR1 and NMUR2 were expressed in the rat hippocampus, and their activation contributed to the excitation of gamma-aminobutyric acid neurons in the hippocampal CA1 zone (31). Neuromedin U is highly expressed in the paraventricular nucleus of the hypothalamus, which participates in stress-related behaviors and regulates the activation of the hypothalamic-pituitary-adrenal axis (27). The NmU gene is located near the clock circadian regulator gene (CLOCK) and is expressed in the suprachiasmatic nucleus, where it helps to regulate the circadian rhythm through rhythm-matched oscillations in its expression level (32, 33). Neuromedin U participates in the regulation of food intake through the brain-gut axis, and intracerebroventricular administration of anti-NMU IgG into rats mediated leptin to induce satiety, decrease food intake, and improve energy consumption (34). Similar studies have shown that an NMUR2 agonist can inhibit food intake and attenuate body weight in diet-induced obese (DIO) rats (35, 36). In addition, peripherally given NMU enhanced glucose tolerance and improved white adipose tissue beiging in DIO mice (37). Neuromedin U is highly expressed in a variety of inflammatory models. It promotes the release of inflammatory factors by binding to receptors on the surface of immune cells, thereby inducing inflammatory reactions (38–42). Neuromedin U activated lung ILC2s by NMUR1, which further promoted IL-17A secretion and IL-17A-dependent γδ T cell amplification, which in turn exacerbated sepsis (43). However, the mechanisms by which NMU affects tumors are relatively complex. For example, NMU expression is significantly reduced or completely silenced in esophageal squamous cell carcinoma and other squamous cell carcinoma cell lines. Further functional tests showed that NMU treatment for cancer cells reduced tumor colony formation (44, 45). Therefore, NMU inhibits the growth of these tumor cells. However, in bladder cancer (46), endometrial carcinoma (47, 48) and breast cancer (49), lung adenocarcinoma (50), NMU overexpression can promote tumor cell growth, enhance tumor formation and metastasis, and increase tumor drug resistance. The latest study found for the first time that NMUR2 activation promoted signaling in colorectal cancer (CRC) cells, and that NMU improves the mobility and invasiveness of NMUR2-positive CRC cells (51). The specific mechanisms behind these differing effects are not clear, but may be related to the tumor type, positive, and negative regulation of oncogenes, and the tumor microenvironment.

### NMU Research Contributions Around the World

The WoS database contains all documents published about NMU since 1987. The United States had the highest number of publications, followed by Japan. Accordingly, of the top 10 institutions, four were from Japan and four were from the United States; among them, the top three most highly cooperative institutions were National Cerebral and Cardiovascular Center Research Institute (Japan), Miyazaki University (Japan), and University of Texas Medical Branch (USA). Miyazato Mikiya, Mori Kenji, and Kangawa Kenji were identified as outstanding scientific leaders in this field. These results emphasize the major contributions of Japan and the United States to this field. Japan was the first country to discover and synthesize NMU, and has the most research institutions currently conducting research on the topic. Although the United States was approximately 20 years behind Japan in contributing to this research field, it currently maintains a dominant position in terms of number of publications, citation frequency, and H-index. This could be due to many factors, including the research institutions, the strength of the publishing journal, academic influences, funding support, and language differences. China is the only developing country in the top five, which included one North American country, one Asian country, and two European countries. In contrast, China ranked third in the number of publications, and ranked fifth in citation frequency and H-index. This could be due to a lack of close team cooperation or a language disadvantage. Although the trend appears to be changing, English is more difficult to publish in for Chinese researchers.

NMU-related studies have been published in a variety of high-impact journals, such as Cell (IF = 41.582), Nature (IF = 49.962), Cell Metabolism (IF = 27.287), Blood (IF = 22.113), and Immunity (IF = 31.745). This indicates advanced research quality in the NMU field, highlighting its importance in biomedicine. Four NMU studies were highly cited, three of which were published in Nature and describe NMU's regulation of ILC2s involved in the inflammatory response. Independent studies showed that NMU was produced by cholinergic neurons in the gastrointestinal tract and lungs of mice, and are adjacent to ILC2s, which selectively express NMUR1. The activated ILC2s proliferate rapidly and release T helper 2 (Th2)-like inflammatory factors, such as interleukin (IL)5, IL13, and amphiregulin. When NMUR1 is knocked out, the Th2 inflammatory response is attenuated (38, 40, 42), suggesting that the NMU-NMUR1 axis plays a key role in the inflammatory response, and confirming that neuropeptides that bypass traditional immunological mechanisms are involved in the regulation of specific immune responses. Exploring the relationships between neuroimmunity, inflammation, allergy, and other diseases has become a promising research direction in the field.

It is worth noting that most of the functions of NMU are performed via NMUR1 and NMUR2. However, other receptors can play regulatory roles as well, such as MAS-related GPR family member X2 (MRGPRX2) (52); NMUR2S (a splice variant of NMUR2) is thought to have a negative regulatory effect on NMU (53). Interestingly, NMUR1 and NMUR2 have been reported to be heterodimers of the growth hormone secretagogue receptor 1b (GHSR1b) and the neurotensin receptor 1 (NTSR1) (54). When considering NMU as a target for disease treatment, mutual cooperation or antagonism between its multiple receptors should be considered.

### Advantages and Limitations of the Study

Based on the advantages of bibliometric systematization and quantification, this study combined the VOSviewer software to draw a scientific map of the field of NMU in order to evaluate the global distribution characteristics, research hotspots, and frontier trends. Our study will help researchers and institutions to better grasp the research dynamics, so as to promote the research progress and translation of results in the field. However, the study also has some limitations. Firstly, the data cannot be analyzed comprehensively across databases, and therefore we have not combined our dataset with data from the PubMed, Scopus, and EMBASE databases for integrated analysis. Moreover, NMU related datas were retrieved from the WoS database, which is recognized as the best database for bibliometric analysis. The data are objective and comprehensive, reflecting the current NMU research situation and its trends. Secondly, this research lacked depth compared with basic research, but this quantitative approach presented a more intuitive overview of topical issues and emerging trends in the field. Thirdly, owing to the short publication time of recently published high-quality literatures, the citation frequency is not high, and the literature quality level will have a certain deviation. In addition, peer review of conference abstracts is considered inferior to that of published papers and there may be positive results bias; on the other hand, conference abstracts are timely and innovative.

Despite these limitations, we believe that the bibliometric method is worth adopting in order to guide the selection of basic and clinical research directions, and improve the quality of future research within the NMU field.

## Conclusions

Bibliometric and visualization analysis of global data on NMU publications in the WoS database revealed that NMU research is continually expanding, and that researchers in the United States and Japan have played key roles in advancing the field. Many NMU studies have been published in influential journals. Neuromedin U plays a variety of roles in the body, and in the future, its roles in metabolism, obesity, inflammation, and cancer in particular will continue to command the attention of scholars. Owing to its promise as a therapeutic target, NMU is worthy of thorough development.

## Data Availability Statement

The raw data supporting the conclusions of this article will be made available by the authors, without undue reservation.

## Author Contributions

XQ and CZ performed to designed this research. PL and YW analyzed the data and worked on pictures and forms. YA and JX revised the draft manuscript. CZ oversaw the research, monitored the data collection, and revised the draft manuscript. All authors contributed to the final article and approved the submitted version.

## Funding

This project was funded by National Natural Science Foundation of China (No: 81670914, 81870707, 81970865, and 82171119) and Shanxi Province Science Foundation for Youths (No: 201801D221412).

## Conflict of Interest

The authors declare that the research was conducted in the absence of any commercial or financial relationships that could be construed as a potential conflict of interest.

## Publisher's Note

All claims expressed in this article are solely those of the authors and do not necessarily represent those of their affiliated organizations, or those of the publisher, the editors and the reviewers. Any product that may be evaluated in this article, or claim that may be made by its manufacturer, is not guaranteed or endorsed by the publisher.
